# Supplementation with postbiotic from *Bifidobacterium Breve* BB091109 improves inflammatory status and endocrine function in healthy females: a randomized, double-blind, placebo-controlled, parallel-groups study

**DOI:** 10.3389/fmicb.2023.1273861

**Published:** 2023-11-23

**Authors:** Diana Elena Motei, Beyda Beteri, Piril Hepsomali, George Tzortzis, Jelena Vulevic, Adele Costabile

**Affiliations:** ^1^School of Life and Health Sciences, University of Roehampton, London, United Kingdom; ^2^School of Psychology and Clinical Language Sciences, University of Reading, Reading, United Kingdom; ^3^veMico Ltd., Reading, United Kingdom

**Keywords:** chronic inflammation, sex hormones, postbiotics, bifidobacteria, intestinal epithelial barrier function, cell-wall polysaccharides, microbial associated molecular patterns

## Abstract

This study evaluated the effects of dietary supplementation with a postbiotic extract of *Bifidobacterium breve* BB091109 on pro-inflammatory cytokines levels and markers of endocrine function. A prospective, double-blind, placebo-controlled, randomized, single-centered, parallel study was conducted on a group of 40–55-year-old females. The study included 30 healthy females, divided into two groups: a supplement (*n* = 20) and a placebo (*n* = 10) groups. Blood and saliva samples were collected at baseline (wk0), after 4 weeks (wk 4) and 12 weeks (12wk) of daily supplementation (500 mg), and 4 weeks (wk 16) after termination of supplementation. The levels of fasting CRP, IL-6, IL-10, TNF-α, IFN-γ, DHEA, estradiol, estriol, progesterone, cortisol and human growth hormone were analysed. The results revealed a significant effect of the 90-day supplementation with *B. breve* postbiotic extract on changes in CRP, IL-6 levels, DHEA, estradiol and estriol. In conclusion, the supplementation with the *B. breve* postbiotic extract improved endocrine function in females over 40 years old and induced protective changes in inflammatory markers. These findings highlight the potential health benefits of this supplementation in promoting hormonal balance and reducing inflammation in this population.

## 1 Introduction

Chronic inflammation has become a significant contributor to various physical and mental health problems, playing a substantial role in global illness and death rates (Netea et al., 2017; Bennett et al., 2018). Normally, inflammation is a temporary reaction to threats that resolves once the threat is eliminated. However, certain factors such as social, psychological, environmental, and biological influences can hinder the resolution of acute inflammation, leading to persistent, non-infectious systemic chronic inflammation (Calder et al., 2013; Straub, 2017). This transition from acute to chronic inflammation can disrupt immune tolerance, impacting tissues, organs, and cellular processes, accelerating the aging process, and increasing the risk of various non-communicable diseases (Kotas and Medzhitov, 2015; Furman et al., 2017; Gisterå and Hansson, 2017). Systemic chronic inflammation can also impair immune function, making individuals more susceptible to infections, tumors, and reduced vaccine responses (Taniguchi and Karin, 2018). Depending on the duration and extent of inflammation, metabolic and neuroendocrine changes occur to conserve energy and support immune activity (Kotas and Medzhitov, 2015; Straub and Schradin, 2016). These changes, known as sickness behaviors, include symptoms like fatigue, disrupted sleep, decreased appetite, and social withdrawal (Straub et al., 2010). In addition to genetic factors and exposure to environmental triggers, two further components have been identified as contributors to chronic inflammatory disorders: an inappropriate increase in intestinal permeability, which may be influenced by the composition of the gut microbiota, and an overactive immune system that disrupts the balance between tolerance and immune responses (Bjarnason et al., 1995; Leclercq et al., 2014; Gonzalez-Gonzalez et al., 2018). The gut microbiota represents a central ecosystem that changes from one site to another (Geuking et al., 2011; Korpela and de Vos, 2018). Its composition is individualized and dynamic, and it depends on age, the influence of diet, environmental conditions of the intestine, lifestyle, and other host related factors (Derrien and van Hylckama Vlieg, 2015). The microbiota contains all three domains of life (i.e., fungi, yeast, protozoa), but consists predominately of anaerobic microorganisms, including thousands of bacterial species and millions of genes (Zoetendal et al., 2008) that play an important role in homeostatic mechanisms leading to the regulation of numerous physiological activities both in health and disease (de Vos et al., 2022). In addition to aiding in the digestion of foods to produce favorable by-products, the gut microbiota also has an important role in the development and function of innate and adaptive immunity (Chen et al., 2017) by essentially establishing a “tolerant” phenotype (Hooper et al., 2012). Reciprocally, the host immune system plays an important role in shaping the gut microbiota (Chen et al., 2017). Due to their proximity, it is essential that the gut microbiota and intestinal immune system tolerate one another in order to enable the continuation of a healthy host-microbe coexistence that maintains intestinal barrier function (Bischoff, 2011). Any disruption in the balance between the gut microbiome and the mucosal immune system will impair the intestinal barrier function and increase the risk of developing various local diseases, immune-mediated disorders, and extraintestinal diseases, characterized by chronic inflammation. Numerous bacterial products regulate intestinal barrier function by activating Toll-like receptors (TLR) and nucleotide-binding and oligomerization domain (NOD)-like receptor (NLR) pathways (Marques and Boneca, 2011; Mu et al., 2015). Microbial associated molecular patterns (MAMPs), like lipopolysaccharide (LPS), flagellin, peptidoglycans, exopolysaccharides, formyl peptides and unique nucleic acid structures, are detected by transmembrane and cytoplasmic pattern recognition receptors (PRRs), initiating conserved signaling cascades that drive stimulatory or regulatory effector responses crucial for host defense (Ali et al., 2020). The healthy intestinal barrier maintains a hypo-reactivity to those MAMPs, while in the underlying immune-stromal-rich layer of lamina propria, the innate immune cells exhibit greater reactivity to commensal and pathogenic microbial ligands (Iwasaki and Kelsall, 2000). Dysbiosis of the gastrointestinal microbiota, characterized by increased LPS concentration, has recently been highlighted as one of the major contributors to intestinal mucosal permeability, induction of innate defenses, and thus an environmental risk factor capable of triggering chronic inflammation (Dorrestein et al., 2014). LPS binding to TLR4 in intestinal cells initiates an inflammatory process that ultimately downregulates the levels of tight junction proteins and favors the translocation of LPS into the systemic circulation, inducing thus local and systemic inflammatory processes that can further affect distant organs, including the lung, liver, brain and skin (Ghosh et al., 2020). The crucial role for the LPS–TLR4 pathway in modulating intestinal barrier integrity has been demonstrated in several studies in experimental models using TLR4 inhibitors or Tlr4-knockout animals (Fort et al., 2005; Peterson et al., 2010; Wang et al., 2015). The administration of the main probiotic genera *Bifidobacterium* and *Lactobacillus* have been reported to lead to an improvement in several factors related to intestinal barrier integrity and inflammation (Zheng et al., 2023). The gut *Bifidobacterium* population is resident within the GI tract throughout our whole lifespan (Asnicar et al., 2017). As one of the first inhabitants of neonatal intestines, bifidobacteria play pivotal roles in the modulation of mucosal physiology and fine-tuning of the host innate and adaptive immune development (Lin et al., 2022) and are associated with immune well-being. *Bifidobacterium* spp. have been reported to suppress levels of proinflammatory cytokines, increase intestinal absorption of electrolytes, repair intestinal permeability, inactivate carcinogens, induce apoptosis, improve T-cell proliferation and cytotoxicity, and modulate natural killer (NK) cell and dendritic cell (DC) interactions, in a strain specific manner (Lee et al., 2017; Wilkins and Sequoia, 2017).

Research to date (Ruiz et al., 2017) has shown that bifidobacteria play a critical role in promoting host immune health by modulating intestinal epithelial and immune cells through the release of various MAMPs, including capsular polysaccharides (CPS) and exopolysaccharides (EPS), during their growth. Those MAMPs crosstalk with PRRs present on the membrane of epithelial/immune cells to configure the cellular structure of the intestinal epithelial barrier (Ruiz et al., 2017). Such polysaccharides have been reported to play an immunomodulatory role (Fanning et al., 2012). EPS from *Bifidobacterium longum* BCRC 14634 was able to stimulate macrophages J774A to produce increased level of anti- inflammatory cytokine IL-10 and to lower levels of pro-inflammatory TNF-α after LPS challenge (Wu et al., 2010), while the EPS-producing *B. breve* UCC2003 significantly decreased the production of pro-inflammatory cytokines both *in vitro* and *in vivo* (Hughes et al., 2017). Moreover, *B. breve* UCC2003 producing EPS was required to reduce pathological epithelial cell shedding in a mouse model, as EPS- knock out isogenic strain could not (Salazar et al., 2014). Using a mouse model to compare the effects of EPS-producing wild-type strain with its isogenic EPS negative mutant, it has been reported that the protection offered by *B. longum* 35,624 against the occurrence of colitis and respiratory allergy symptoms was dependent on the presence of EPS (Schiavi et al., 2016). More recently, a polysaccharide synthesized by *B. bifidum*, consisting of β-glucans/galactans was found to be the key in the induction of Treg cells. The polysaccharide efficiently repeated the activity of whole bacteria and was demonstrated to act via dendritic cells through TLR2 -mediated mechanism (Verma et al., 2018). Overall, it has been postulated that bifidobacterial molecules, based on immune receptor ligand interactions and downstream signaling events, regulate T-cell responses and strengthen immune tolerance to the existing colonic environment (including the microbiota and its metabolites), preventing thus colonic tissue damage and inflammation (Knoop et al., 2017). Temporal variance as a feature of the microbiome has been discussed before (Bastiaanssen et al., 2020) and the term volatility has been scarcely used in the context of the microbiome. Recently, some studies have shown that volatility may be related to stress although the impact of stress on microbial volatility measures remains unknown (Clooney et al., 2020). Furthermore, given that re-enacting the gut microbiome’s signaling function with postbiotics, such as CPS and EPS, rather than maintaining a specific bacterial species composition may be a more reliable and efficient approach to maintain intestinal epithelial barrier function reducing the risk of developing low-grade systemic inflammation and/or chronic inflammatory diseases. To this end, our aim was to conduct a double-blind, placebo-controlled intervention study in healthy females over 40 years old to evaluate the potential of a mixture of exopolysaccharides from *B. breve* BB091109 (that have been shown to act through MAMPs *in vitro* and barrier function based on data and role of TLR2/TLR4 presented in Supplementary Table S1) on markers of chronic systemic inflammation, during and following a 3-month supplementation period.

## 2 Materials and methods

### 2.1 Test product and placebo

Both the test product and the placebo were administered in identical HPMC capsules (size 0) and packaged in identical containers, providing a one-month supply of 30 capsules. The test product, VMK223 (veMico Ltd., UK) consisted of 500 mg of a β-glucans mixture extracted from *Bifidobacterium breve* BB091109 (NCIMB43992). In details, 51.7 g VMK223 was recovered per litre of *B. breve* fermentation, consisting of 74% (w/w) β-glucan mixture, 16% (w/w) other oligosaccharides and 1% (w/w) protein. VMK223 was obtained during the stationary stage of growth in a specially formulated fermentation medium containing 24 g/L yeast extract, 26 g/L soy peptone, 25 g/L lactose, 20 g/L fucose-containing oligosaccharides, 10 g/L glucose, 1 g/L Tween 80, 2 g/L K_2_HPO4, 5 g/L sodium chloride, 3 g/L sodium acetate, 9 g/L bile salts and 20 mM taurocholic acid. Following anaerobic fermentation for 60 h at 37°C, *B. breve* cells were collected through centrifugation and dissolved in 1 N NaOH overnight at room temperature, while the supernatant was kept at 4°C for further treatment. The solution was further centrifuged, and the supernatant collected. The two collected supernatants were further treated with 1 vol 96% cold ethanol overnight and centrifuged to collect the precipitate, three times. All collected precipitates were dissolved in distilled water and heated to 100°C for 5 min. Following overnight dialysis (MWCO 10KDa), the retentate was freeze dried and used to prepare the 500 mg VMK223 containing capsules.

Cellulose microcrystalline (Alfa Aesar, UK), a common excipient in the pharmaceutical industry known for its lack of impact in the colonic environment, was chosen as the placebo. The test product and placebo were taken daily before or together with a meal. Subject’ compliance was followed by daily questionnaires.

### 2.2 Study design and ethical aspects

This study utilized a single-center, double-blind, randomized, placebo-controlled and parallel study design. The protocol adhered to the Helsinki Declaration and received approval from the University of Roehampton Research Ethics Committee (Ethics reference number: LSC 18/274). The study was registered as a clinical trial (ID: NCT04267731).^1^ Participants provided written consent, and the selection process involved a medical interview to determine health status and adherence to the inclusion/exclusion criteria. The primary and secondary outcomes of the study aimed to evaluate the efficacy of a 12-week consumption of 500 mg VMK 223 on plasma markers of chronic low-grade inflammation (Plasma C-reactive protein (CRP), IL-6), plasma inflammatory markers (TNF-α, IFNγ, IL-10), selected saliva hormone levels (oestradiol, DHEA, estriol, progesterone, cortisol) and plasma human growth hormone, respectively. Additionally, product tolerance was assessed for potential adverse events and gastrointestinal (GI) side effects following 12 weeks of intake.

### 2.3 Study participants

**Figure 1:**
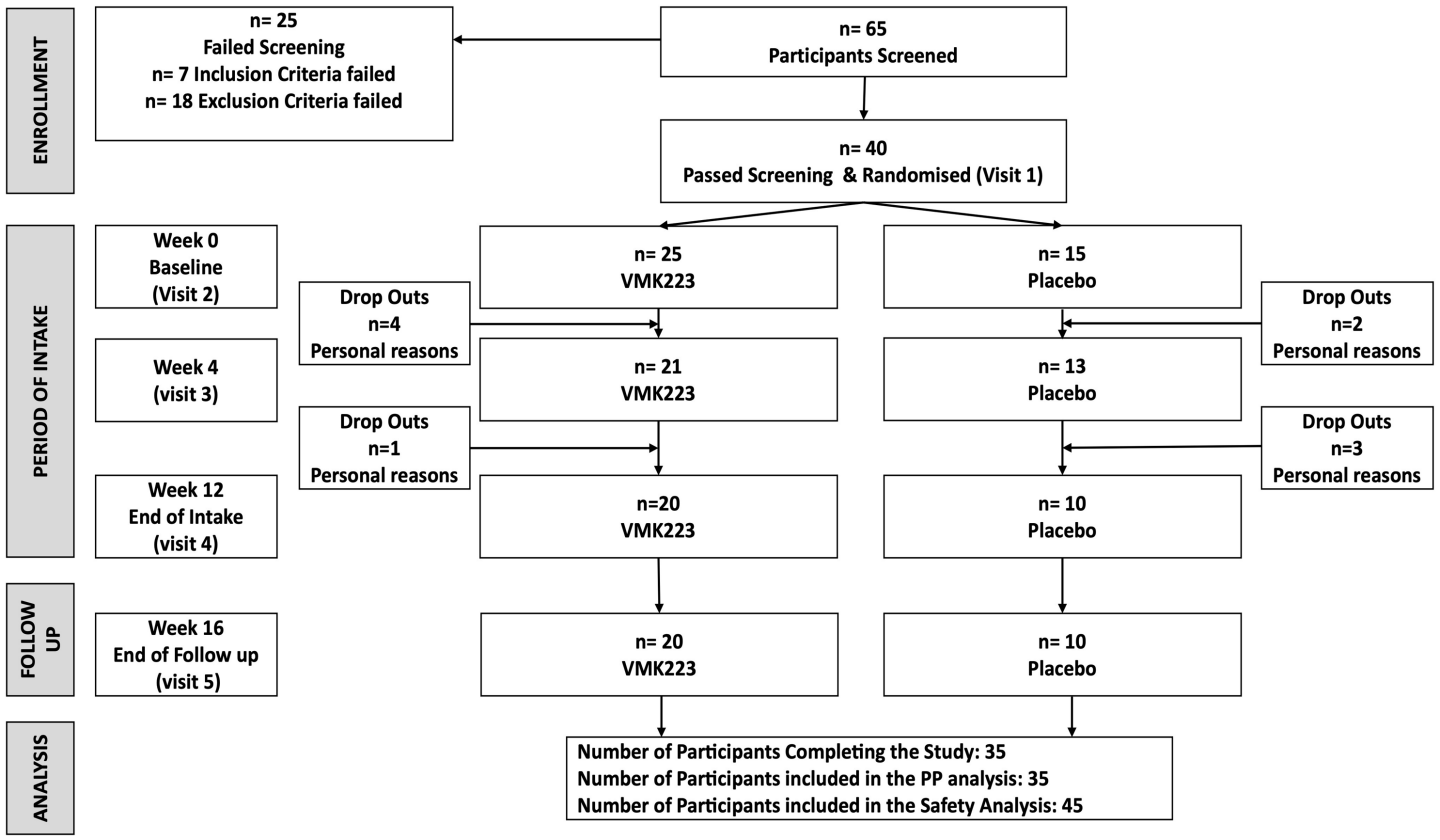
The study flow diagram. VMK223, *Bifidobacterium breve* BB091109 postbiotic; PP, per protocol.

A total of 40 female subjects aged between 40 and 55 years participated in this study (Figure 1). The main exclusion criteria were as follows: significant health problems (e.g., hypercholesterolaemia, diabetes, GI disorders), the use of medications or supplements known to affect mineral or glucose metabolism within the month prior to the study and/or during the study, pregnancy or plans to become pregnant, breastfeeding, hormone replacement therapy, a history of anaphylaxis to food, known allergies or intolerance to foods and/or to the study materials (or closely related compounds) or any of their stated ingredients, ongoing dieting, having lost >5% body weight in the previous year, and abnormal eating behaviour.

Participants were instructed to maintain their usual living habits, and the intake of other products similar to the test product during the study was not allowed. The recruited subjects were then randomized into two groups to receive either VMK223 or an equivalent placebo comparator. The randomization process utilized an Excel-based covariate adaptive program (Kang et al., 2008) to ensure the volunteers were evenly distributed between the two intervention arms.

### 2.4 Study schedule

The total study duration was 16 weeks, comprising a 12-week treatment period and a 4-week follow up period. Additionally, a 2-week run-in period preceded the study, during which volunteers refrained from consuming any probiotic or prebiotic-containing food or drinks. During the run-in period, participants recorded a 4-day food diary, including one weekend day, to assess their habitual diet. The randomization process utilized and Excel-based covariate adaptive program (Kang et al., 2008) to allocate volunteers into two intervention arms: VMK223 or placebo comparator stratified by age, gender, and Body Mass Index (BMI).

Data collection occurred before the first intake of the study product, after 4 weeks of intake, after the 12-week treatment period, and after the 4-week follow-up period. During these assessments, blood, saliva and tolerability data were collected. At the end of the follow-up period, the sustainability of the previously observed effects was evaluated in the test group of subjects who had taken the test product for at least 80% of the 12-week period.

### 2.5 Collection and analysis of blood and saliva samples

Fasted blood samples and saliva were collected from each participant at four time points: baseline (wk 0) before the intervention began, after 4 weeks of product intake, at the completion of the 12-week study treatment period (wk 12), and at the end of the 4-week follow-up period (wk 16).

For blood collection, participants underwent a 12-h fasting period. Trained phlebotomist obtained the blood samples from the participant’s antecubital vein using a 23G butterfly needle (Greiner Bio-One GmbH, Kremsmünster, Austria). Four types of blood tubes were used: one 9 mL K2EDTA tube, one 9 mL Lithium Heparin tube, one 2 mL Sodium Fluoride/Potassium Oxalate tube, and one 2 mL K2EDTA tube (Vacuette®; Greiner Bio-One GmbH, Kremsmünster, Austria). After collection, all samples were immediately placed on ice until centrifugation.

Plasma samples were obtained by centrifugation at 2095 x g for 10 min, dispensed into 1.5 mL microcentrifuge tubes and promptly frozen at −80°C within 1 h from the collection.

### 2.6 Immune inflammatory markers

CRP, IL-6, IL-10, INFγ, TNF-α and human growth hormone (HGH), were measured using commercially available human selected biomarker kits (V-Plex Panel 2 Human Kit and CRP kit, Meso Scale Diagnostics LLC, USA) in accordance with the manufacturer’s recommendations. This ultra-sensitive method has a detection limit of 1.33 pg/mL for CRP, 0.06 pg/mL for IL-6, 0.04 pg/mL for IL-10, 0.37 pg/mL for INF-γ, and 0.04 pg/mL for TNF-α.

Saliva samples were promptly stored in −80°C freezers following collection until further analysis. Participants were instructed to provide fasting saliva samples (approximately 5 mL) between 6 a.m. and 8 a.m. or within 30 min of waking up. Collection was done before eating, drinking liquids, or brushing teeth. The salivary hormones (estriol, oestradiol, DHEA, progesterone, and cortisol) were tested in duplicate using a fully automated enzyme-linked immunosorbent assay (ELISA) platform (Affinity Labs, London UK).

### 2.7 Anthropometric variables

The anthropometric variables monitored throughout the study included height, weight, body mass index and composition, waist and hip circumference and waist to hip ratio as reported by Keleszade et al. (2022).

### 2.8 Assessment of safety and tolerability

Safety and tolerability of the test material was assessed by two methods: (1) monitoring of adverse events during the study through information collected in interviews and questionnaires; and (2) using a daily GI function questionnaire to record details of bowel habits including stool frequency and consistency (Bristol stool scale), stomach or intestinal bloating, abdominal pain, incidence, and frequency of flatulence.

### 2.9 Statistical analysis

The study was powered to provide 80% statistical power (MGH Biostatistics Hedwig Software) based on a treatment difference in C-Reactive Protein level (1.5 times the standard deviation), based on data from a previous VMK223 dose response study (Costabile et al., unpublished data). Given these calculations, 40 participants (to allow for 25% attrition due to the longer study period) were required to detect a treatment difference at a two-sided 0.05 significance level.

All baseline endpoints from the blood and saliva analysis were analysed using descriptive statistics and compared between groups by using independent samples *t*-tests. The location and scale statistics of all the baseline demographic characteristics and parameters were calculated, including the arithmetical mean, standard deviation and 95% confidence interval for mean (minimum, and maximum). From the calculated values of skewness and kurtosis it was concluded that all the parameters could be regarded as (approximately) normally distributed.

Data from all continuous endpoints were analyzed by using separate 2 × 4 repeated measures of analyses of variance (ANOVA) with Treatment (VMK223 vs. placebo) as the between-subject factor and Time (baseline, after 4 weeks of supplementation, after 12 weeks of supplementation, and after 4 weeks of terminating the supplementation) as the within-subject factor. Significant interactions were subsequently analyzed using Tukey’s post-hoc test. The reliability of assumptions of this statistical test was checked using the Shapiro–Wilk test for normality, and Levene’s test for the homogeneity of variance. Further, the effect size was estimated by eta-squared statistics (*η*2). Values equal to or greater than 0.01, 0.06, and 0.14 indicated a small, moderate, and large effect, respectively. In order to adjust for multiple testing, the Bonferroni method was applied.

Additionally, intra-individual mean changes of (i) CRP and (ii) IL-6 (comparing T0 versus T4, T12, and T16), were evaluated using paired-samples *t* tests. The mean outcomes in the VMK223 and placebo groups were compared using the independent samples *t*-tests. The primary outcomes and test hypotheses of the trial were based on the comparison of the VMK223 and placebo groups with respect to the change (T12–T0) of: (i) CRP, and (ii) IL-6.

The significance threshold was set at *p* < 0.05. All statistical analyses were performed according to the principles of ICH (International Conference on Harmonization) guideline E9 “Statistical Principles for Clinical Trials” using JASP software (Version 0.17.1, JASP Team, Netherlands).

## 3 Results

### 3.1 Baseline characteristics of participants

Participants (*n* = 40) were randomly allocated to either the test group (VMK) (*n =* 25) or the placebo group (*n =* 15). At baseline, there were no significant differences between the two groups in terms of age, body weight, systolic and diastolic blood pressures, and all the selected fasting blood and saliva parameters (Table 1). Throughout the study, all recruited participants completed the trial, and no adverse events were reported. Both products were well tolerated by the participants.

**Table 1:** Demographic and baseline fasting blood and saliva characteristics of all study participants at the beginning of the study.

	VMK223 group (*n* = 20)		Placebo group (*n* = 20)		*p*-value*
	Mean (SD)	Min, Max^$^	Mean (SD)	Min, Max^$^	
Age (years)	48.00 (4.5)	40, 59	48.35 (4.32)	43, 57	0.749
Weight (kg)	54.45 (5.25)	44.3, 70.5	56.73 (4.46)	48.1, 65.3	0.067
Systolic bp (mmHg)	117.61 (13.87)	98, 140	114.52 (11.41)	95, 140	0.336
Diastolic bp (mmHg)	71.79 (10.29)	48, 95	69.35 (9.32)	46, 91	0.326
CRP (mg/L)	5.09 (1.80)	4.31, 5.89	5.10 (1.82)	3.98, 6.23	0.997
TNF-α (pg/ml)	7.72 (1.37)	7.12, 8.32	7.80 (1.10)	7.12, 8.48	0.874
IL-6 (pg/ml)	3.92 (0.68)	3.62 4.21	4.09 (0.52)	3.78, 4.42	0.457
IL-10 (pg/ml)	2.39 (0.85)	2.02, 2.76	2.27 (0.94)	1.69, 2.85	0.724
IFN-γ (pg/ml)	138.85 (23.67)	128.48, 149.23	142.03 (38.17)	118.34, 165.67	0.782
Progesterone(pg/mL)	88.32 (14.08)	82.15, 94.49	99.30 (16.34)	79.17, 99.43	0.866
Oestradiol (pg/ml)	35.01 (12.57)	29.50, 40.52	31.00 (12.18)	23.45, 38.55	0.413
Estriol (pg/ml)	2.04 (0.17)	1.96, 2.11	2.16 (0.18)	2.05, 2.27	0.074
DHEA (ng/ml)	0.81 (0.26)	0.69, 0.92	0.70 (0.18)	0.59, 0.81	0.263
Cortisol (ng/ml)	53.95 (6.25)	51.21, 56.69	53.50 (8.33)	48.34, 58.66	0.868
HGH (ng/ml)	1.34 (0.57)	1.09, 1.59	1.54 (0.55)	1.19, 1.88	0.369

$, 95% Confidence Interval for Mean. *p-values for independent *t*-test comparisons between values in the VMK223 group and the placebo group. For a significant difference by *t*-test, *p* < 0.05. bp, blood pressure; CRP, Plasma C-reactive Protein; DHEA, dehydroepiandrosterone; HGH, human growth hormone.

Daily GI function questionnaires showed no significant effects of time or intervention on the bowel function, mood or dietary intakes following daily supplementation with VMK223 and placebo (data not shown).

### 3.2 Effect of VMK223 on fasting plasma inflammatory markers and salivary hormones

**Figure 2:**
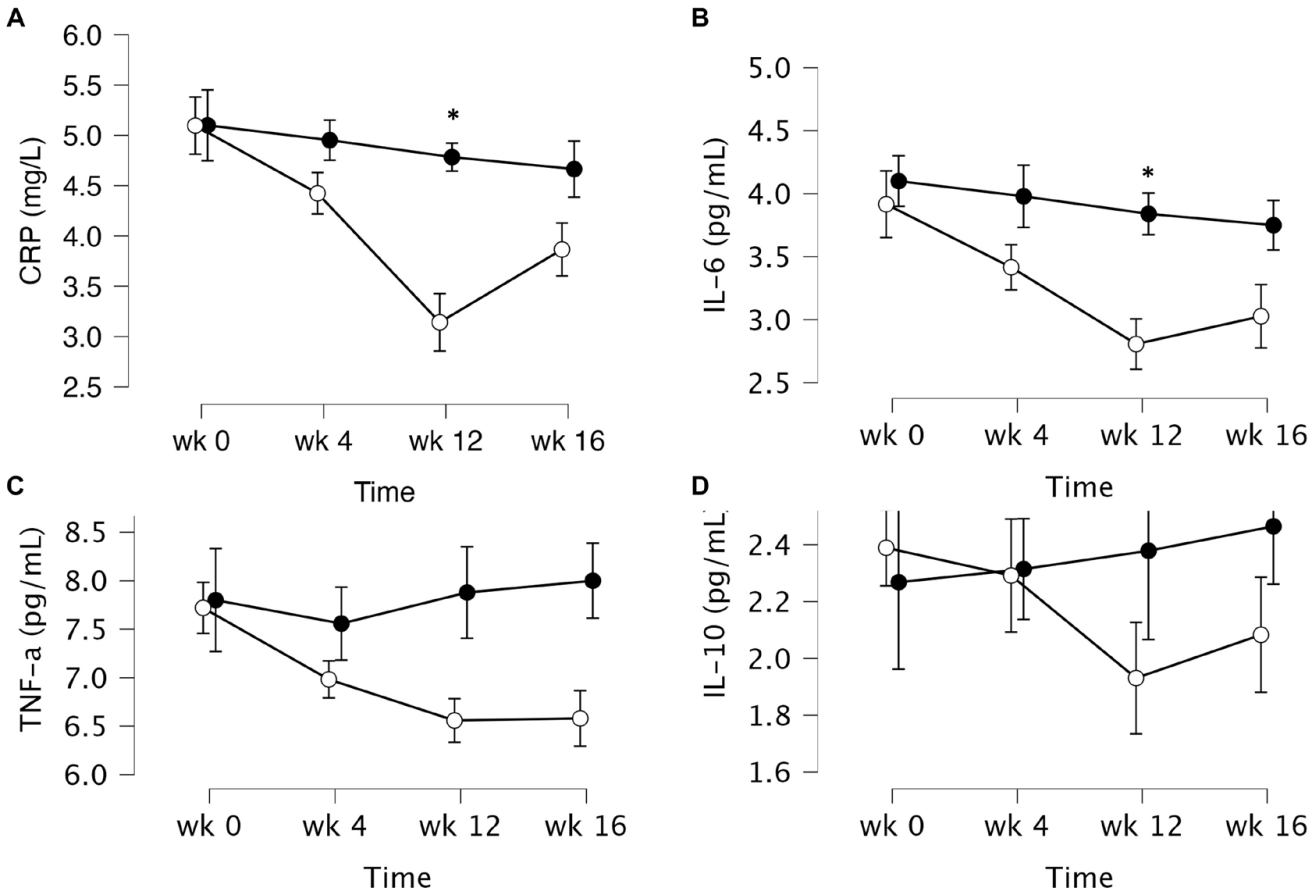
Mean ± SD values of fasting plasma levels of select inflammatory markers after 12 weeks of supplementation with a lyophilizate *Bifidobacterium breve* BB091109 postbiotic (VMK223): **(A)** Plasma C-reactive protein (CRP), **(B)** IL-6, **(C)** TNF-α and **(D)** IL-10. Black circles, placebo group (*n* = 10); white circles, VMK223 group (*n* = 20). wk 0, Baseline; wk 4, following 4 weeks of supplementation; end of treatment with wk 12, end of treatment; wk 16, end of follow up period. *Significant difference versus the placebo at the particular time point.

**Figure 3:**
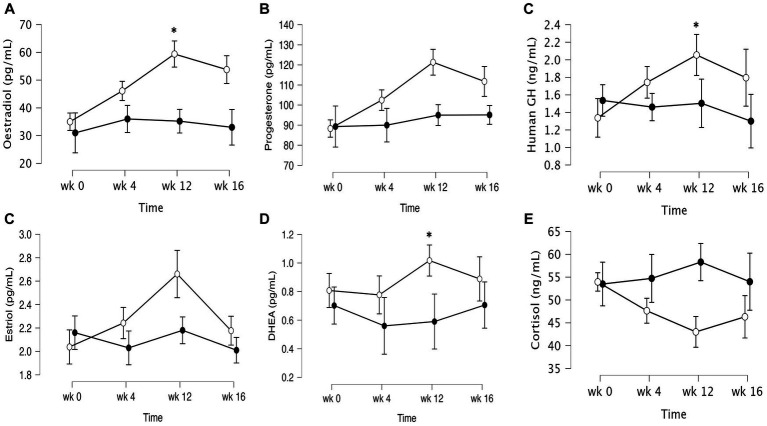
Mean ± SD values of fasting levels of selected after 12 weeks of supplementation with a lyophilizate *Bifidobacterium breve* BB091109 postbiotic (VMK223): **(A)** Oestradiol, **(B)** Estriol, **(C)** progesterone **(D)** human growth hormone, **(E)** cortisol, **(F)** DHEA. Black circles, placebo group (*n* = 10); white circles, VMK223 group (*n* = 20). wk 0, Baseline; wk 4, following 4 weeks of supplementation; end of treatment with wk 12, end of treatment; wk 16, end of follow up period. *Significant difference versus the placebo at the particular time point.

The data of plasma and salivary biomarkers during the study period are shown in Table 2. The statistical analysis (ANOVA) revealed a significant effects of supplementation group, supplementation time, and their interaction for several biomarkers. For the plasma biomarkers, significant effects of the supplementation group were observed on IL-6, DHEA, oestradiol, estriol, progesterone and cortisol levels. Supplementation time had a significant effect on CRP, TNF-α, IL-6, progesterone, oestradiol and estriol levels. Additionally, the interaction between supplementation group and time showed significant effects on CRP, TNF-α, IL-6, IL-10, progesterone, oestradiol, estriol, cortisol and human growth hormone levels. Post-hoc analysis further examined specific differences between the groups and time points for the significant biomarkers. Notable, at the end of the supplementation period with VMK223, CRP levels were significantly lower than at baseline (*p* < 0.001) and lower than the placebo group at baseline (*p* = 0.043) and end of supplementation (*p* < 0.001) (Figure 2). TNF-a levels at the end of the supplementation period with VMK223 were significantly lower than at baseline (*p* < 0.001), but not significantly different from the placebo group levels at baseline (*p* = 0.293) and end of supplementation (*p* = 0.226) (Figure 2). IL-6 levels at the end of the supplementation period with VMK223 were significantly lower than at baseline (*p* < 0.001) and lower than the placebo group at baseline (*p* = 0.001) and end of supplementation (*p* = 0.017) (Figure 2). Regarding hormone data, progesterone levels at the end of the supplementation period with VMK223 were significantly higher than at baseline (*p* < 0.001) and higher than the placebo group at baseline (*p* < 0.001) and end of supplementation (*p* = 0.003) (Figure 3). Oestradiol and estriol levels were significantly higher at the end of the supplementation period with VMK223 than at baseline (*p* < 0.001 for both), and higher than the placebo group levels at baseline (*p* < 0.001 and *p* = 0.002, respectively) and end of supplementation (*p* = 0.003 and *p* = 0.004, respectively) (Figure 3). HGH levels at the end of the supplementation period with VMK223 were significantly higher than at baseline (*p* < 0.001) but not significantly different from the placebo group levels at baseline (*p* = 0.171) and end of supplementation (*p* = 0.118) (Figure 3). Cortisol levels at the end of the supplementation period with VMK223 were significantly lower than at baseline (*p* < 0.001) and lower than the placebo group levels at baseline (*p* = 0.038) and end of supplementation (*p* < 0.001) (Figure 3). At the end of the follow up period, 4 weeks after the termination of the supplementation with VMK223, the levels of IL-6 (*p* = 0.736), IL-10 (*p* = 0.913), TNF-a (*p* = 1.000), DHEA (*p* = 0.203), oestradiol (*p* = 0.452), DHEA (*p* = 0.792), human growth hormone (*p* = 0.661) and cortisol (*p* = 0.824) were not significantly different from the levels at the end of the supplementation period. However, the levels of CRP were significantly higher (*p* < 0.001) and the levels of estriol were significantly lower (*p* < 0.001) than the levels at the end of the supplementation period.

**Table 2:** Two-factor ANOVA with repeated measures (2 groups × 4 time points) of the fasting plasma levels of inflammatory markers and fasting saliva levels of selected hormones before and after 12 weeks of supplementation with VMK223.

	Effect	*F*	df	*p*-value	Effect size (*η*^2^)	Post-hoc outcome
CRP	GR	1.702	1, 28	0.203	0.048	
	RM	25.925	2, 57	<0.001^**^	0.064	B, I > E, F
	GR * RM	12.356	2, 57	<0.001^**^	0.031	VE < VB, PB, PE
TNF-α	GR	2.966	1, 28	.096	0.080	
	RM	5.474	3, 70	0.002^**^	0.021	B > I, E, F
	GR * RM	8.718	3, 70	<0.001^**^	0.034	VE < VB
IL-6	GR	6.221	1, 28	0.019^*^	0.125	V < P
	RM	14.312	2, 63	<0.001^**^	0.095	B, I > E; B > F
	GR * RM	4.511	2, 63	0.012^*^	0.030	VE < VB, PB, PE
IL-10	GR	0.336	1, 28	0.567	0.010	
	RM	1.070	3, 84	0.367	0.005	
	GR * RM	3.438	3, 84	0.020^*^	0.017	VE < VB
IFN-γ	GR	0.305	1, 28	0.585	0.008	
	RM	1.284	2, 65	0.286	0.011	
	GR * RM	2.399	2, 65	0.091	0.021	
Progesterone	GR	7.284	1, 28	0.012^*^	0.111	V > P
	RM	13.239	3, 84	<0.001^**^	0.129	B, I < E; B < F
	GR * RM	5.930	3, 84	0.001^**^	0.058	VE > VB, PB, PE
Oestradiol	GR	8.841	1, 28	0.006^**^	0.165	V > P
	RM	12.996	3, 84	<0.001^**^	0.083	B < I, E, F; I < F
	GR * RM	7.816	3, 84	<0.001^**^	0.050	VE > VB, PB, PE
Estriol	GR	6.173	1, 28	0.019^*^	0.059	V > P
	RM	7.779	2, 64	<0.001^**^	0.129	B, I, F < E
	GR * RM	4.834	2, 64	0.008^**^	0.080	VE > VB, PB, PE
DHEA	GR	GR	1, 28	0.001^**^	0.129	V > P
	RM	RM	3, 84	0.232	0.028	
	GR * RM	1.773	3, 84	0.159	0.034	
Cortisol	GR	10.978	1, 28	0.003^**^	0.139	V < P
	RM	1.317	2, 59	0.276	0.019	
	GR * RM	5.411	2, 59	0.006^**^	0.079	VE < VB, PB, PE
HGH	GR	5.289	1, 28	0.029	0.059	
	RM	2.451	2, 63	0.088	0.045	
	GR * RM	3.494	2, 63	0.032^*^	0.065	VE > VB

GR, treatment; RM, repeat measure; V, VMK223 group; P, placebo group; B, baseline; I, week 4; E, end of treatment; F, end of follow up. Significant difference at * *p* < 0.05 and ** *p* < 0.01.

### 3.3 Effect of VMK223 on markers of chronic low-grade inflammation

**Figure 4:**
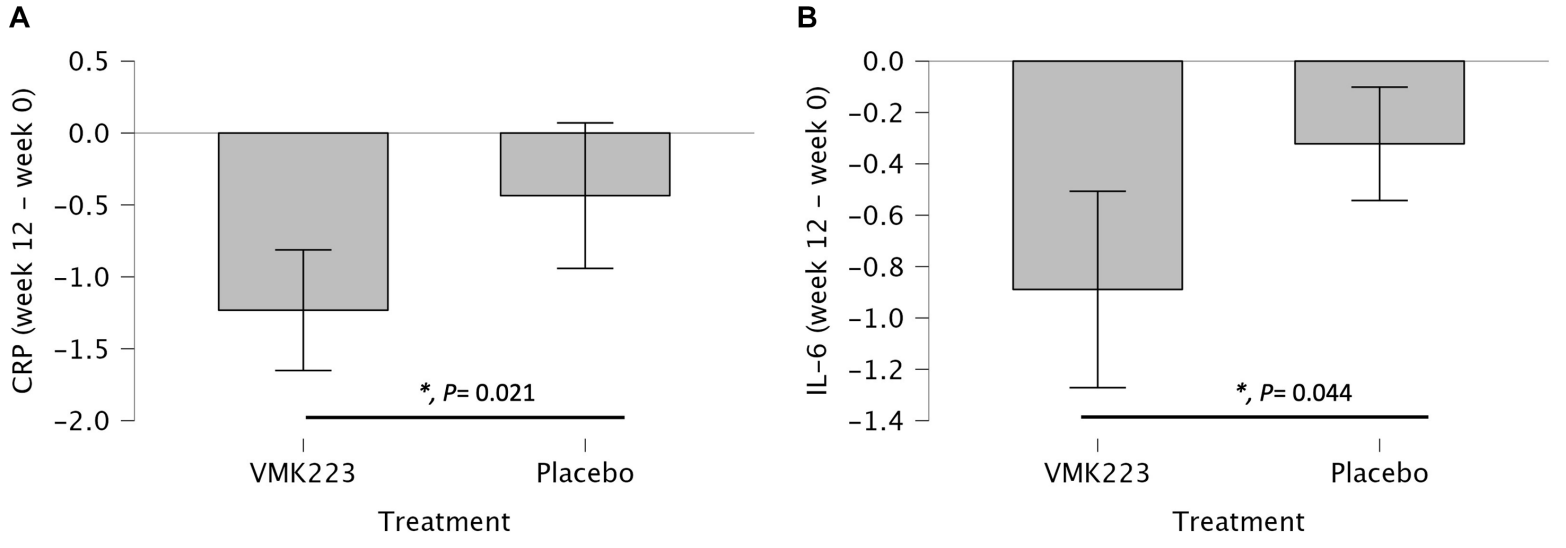
Changes in Plasma C-reactive protein (CRP) (mg/L) and IL-6 (pg/mL) in individuals *Bifidobacterium breve* BB091109 postbiotic (VMK223) preparation or placebo. **(A)** Changes in CRP. **(B)** Changes in IL-6. Changes in parameter values from baseline are shown in mg/L (CRP) or pg/mL (IL-6). Data are expressed as Mean ± SD. *p-values for *t*-test comparisons between values in the test group and the placebo group; *p < 0.05.

In the test group, CRP levels showed a significant reduction after 4 weeks (*p =* 0.002) and 12 weeks (*p* < 0.001) of supplementation, as well as 4 weeks (*p* < 0.001) after the termination of supplementation, compared to baseline. In contrast, the placebo group exhibited no significant difference in CRP levels from baseline at 4 weeks (*p =* 1.000), 12 weeks (*p* = 0.852) and 4 weeks (*p* = 0.531) post supplementation (Table 3). Between-group comparisons showed that CRP values were significantly lower in the test group than in the placebo group after 12 weeks of supplementation (*p =* 0.001) (Table 3). Figure 4 illustrates that at the end of the 12-week supplementation period, the test group had significantly lower CRP levels compared to the placebo group (*p* = 0.021). Similarly, IL-6 levels in the test group demonstrated a significant reduction after 4 weeks (*p =* 0.009) and 12 weeks (*p* < 0.001) of supplementation, as well as 4 weeks (*p* < 0.001) after the termination of supplementation compared to baseline. Conversely, the placebo group exhibited no significant difference in IL-6 levels from baseline at 4 weeks (*p =* 0.998), 12 weeks (*p* = 0.875) and 4 weeks (*p* = 0.607) post supplementation (Table 3). Comparing the two groups, IL-6 values were significantly lower in the test group than in the placebo group after 4 weeks (*p =* 0.031), 12 weeks (*p =* 0.004), as well as 4 weeks (*p* = 0.034) after the termination of supplementation (Table 3). Figure 4 demonstrates that at the end of the 12-week supplementation period, the test group had significantly lower IL-6 levels compared to the placebo group (*p* = 0.044, respectively). At the end of the 12-week supplementation period, the test group demonstrated a substantial reduction in both CRP and IL-6 levels compared to the placebo group, with the reduction being 4-fold greater for CRP and 4.7-fold greater for IL-6.

**Table 3:** Concentration of fasting plasma CRP and IL-6 before and after supplementation with a *Bifidobacterium breve* BB091109 postbiotic (VMK223) extract (Mean ± SD).

	VMK223 group (*n* = 20)		Placebo group (*n* = 20)		Between group *p*-value*
	Mean ± SD	*p*-value	Mean ± SD	*p*-value	
CRP (mg/L)					
Baseline	5.09 ± 1.80		5.10 ± 1.82		0.997
Week 4	4.42 ± 1.62	0.002*	4.95 ± 1.74	1.000	0.418
End of treatment (12wk)	3.14 ± 0.94	<0.001**	4.78 ± 1.59	0.852	0.001^$^
End of follow up (16wk)	3.87 ± 1.29	<0.001**	4.67 ± 1.62	0.531	0.153
IL-6 (pg/ml)					
Baseline	3.92 ± 0.68		4.09 ± 0.52		0.457
Week 4	3.42 ± 0.69	0.009*	3.98 ± 0.49	0.998	0.031^*^
End of treatment (12wk)	2.81 ± 0.94	<0.001**	3.84 ± 0.64	0.875	0.004^*^
End of follow up (16wk)	3.03 ± 0.93	<0.001**	3.75 ± 0.59	0.607	0.034^*^

*p*-values for *t*-test comparisons between values in the VMK223 group and the placebo group.

*p < 0.05, **p < 0.001.

## 4 Discussion

In this study, we investigated the effects of VMK223, a postbiotic mixture of exo- and capsular- polysaccharides derived from *Bifidobacterium breve* BB091109, consisting mainly of β-glucan, on plasma markers of systemic inflammation and sex hormones in healthy females over 40 years old who were not receiving hormonal therapy. The daily oral supplementation with VMK223 (500 mg daily) resulted in a significant reduction in markers of systemic inflammation (CRP, IL-6, TNF-α) after 4 weeks of supplementation, which was sustained throughout the 12-weeks treatment period and the 4-week follow-up period. This reduction in systemic inflammatory markers was accompanied by a significant decrease in cortisol levels, a hormone associated with increased energy-expenditure and immune system activation (Roney and Simmons, 2015). Cortisol increase is known to lead to a significant decrease of female sex hormones estrogen, oestradiol, progesterone, and DHEA (Roney and Simmons, 2015). Current data support sexual dimorphism of the gut microbiota in animals (McGee and Huttenhower, 2021). Moreover, mucosal immune function and susceptibility to chronic inflammation differs between sexes (Casimir and Duchateau, 2011). Those distinct differences in the male and female gut microbiota, for both animal and human models, inevitably generate differences in metabolic processes and therefore, differences in dysbiosis and the protection or susceptibility to chronic systemic inflammation (Casimir and Duchateau, 2011). We have therefore selected only female participants to overcome potential sexual dimorphism issues and chosen the perimenopause stage, as the endocrine transition during this life stage has been associated with reduction in the integrity of the intestinal barrier and a rise in chronic low-grade inflammation (Brettle et al., 2022). The pathogenesis of numerous chronic inflammatory diseases (CIDs) involves complex interactions among genetic predisposition, environmental triggers including alterations in the gut microbiome, gut permeability, antigen trafficking and immune activation (Fasano, 2020). At the forefront of this interplay are the biological interfaces that separate our bodies from the external environment. Of these interfaces, the human intestine represents the largest and most critical interface. Through tightly packed epithelial cells, the intestinal mucosa interacts with various environmental factors, such as microorganisms, nutrients, and antigens to regulate molecular traffic between the gut and submucosa, impacting immune responses and tolerance (Yoo et al., 2020; de Vos et al., 2022). The gut microbiota plays a vital role in maintaining the health of the host and it influences various physiological processes, including digestive function, pathogen resistance, intestinal permeability, endocrine function, and immune stimulation. The influence of the gut microbiota on human health extends beyond metabolic activities. Microbial translocation, a process where disruptions in the gut epithelial barrier allow microbes and their products to enter the systemic circulation, plays a role in several diseases (de Vos et al., 2022). The activation of TLRs by MAMPs has been shown to contribute to the development of chronic systemic inflammation. Dysbiosis in the gut microbiome can lead to compromising the epithelial barrier and causing the translocation of luminal contents, triggering an inflammatory response (Salvo-Romero et al., 2020). Depending on the host’s genetic makeup, activated T cells may remain in the gastrointestinal tract, leading to CID of the gut, or migrate to other organs, causing systemic CIDs. It is well known that certain bacterial species, such as *Akkermansia muciniphila*, *Bifidobacterium* spp., and *Lactobacillus* spp. have been shown to improve intestinal barrier integrity and reduce inflammation (Jian et al., 2023). Probiotic administration has shown promising results in enhancing intestinal barrier integrity, reducing translocation of LPS, alleviating low-grade systemic inflammation, and improving endocrine function and immune tolerance (Plaza-Diaz et al., 2014; Zheng et al., 2023). Additionally, *B. breve* has been reported to modulate the expression of TLRs, normalizing TLR4 expression, enhancing TLR2 expression, and reducing the expression of pro-inflammatory cytokines, including IL-1 beta, IL-6, and tumor necrosis factor alpha in an animal model of necrotizing enterocolitis (NEC) (Wong et al., 2019). The current findings are also in line with results from experimental models that have shown that the administration of specific *Bifidobacterium* strains, such as *B. breve* M-16 V, reduced the severity of a condition characterized by intestinal inflammation and damage (Satoh et al., 2016; Wong et al., 2019). It has been demonstrated that viability is not a pre-requisite for the health benefits associated with probiotic administration. Postbiotics have emerged as a new class of bacteria derived effector molecules that are either produced by live bacteria or are released after bacterial cell lysis (Vinderola et al., 2022). Postbiotics must be derived from a well-defined microorganism or combination of microorganisms for which genomic sequences are known and prepared using a delineated technological process of biomass production and inactivation, which can be reliably reproduced (Tsilingiri and Rescigno, 2013; Vinderola et al., 2022). A postbiotic may be inanimate intact cells or may be structural fragments of the microorganisms, such as cell walls. Among the various potential cell wall postbiotics, particular attention is given to CPS and EPS. Recent work has described the unique and potent antigen polysaccharide A (PSA) synthesized by *Bacteroides fragilis* and its involvement in promoting the differentiation of mouse Treg cells, through the expression of TLR2 on both DC and T cells (Mazmanian et al., 2005, 2008). It was also demonstrated that a critical step defining the pro-tolerance phenotype and differentiation of Treg cells was the recognition of PSA by plasmacytoid DCs. Similarly, *Bifidobacterium* spp. derived EPS has been reported to play an immunomodulatory effect (Fanning et al., 2012). Overall, the cell wall polysaccharides, CPS and EPS, from *Bifidobacterium* spp. and some other Gram+ commensal bacteria are recognized by the TLR2 receptor, although they may also bind directly to other PRRs such as C-type lectin receptors, e.g., dectin-1, dectin-2, or mannan receptors, affecting the signaling pathway induced by bacteria. Recent research has also reported that such polysaccharides from Gram+ beneficial commensals can also be recognized by the TLR4 receptor and act as an LPS-antagonist (Zheng et al., 2020). Nevertheless, the chemical composition and structure of EPS defines its immunomodulatory properties and has been shown to depend on genetics (bacterial species) and environmental (growth conditions) factors. Following fermentation of *B. breve* BB091109 under different conditions of stress (pH, bile salts, O2 levels), carbon sources and ethanol extraction, VMK223 was selected for this study following *in vitro* screening with HEK (Human Embryonic Kidney) 293 cell line, based on its increased ability to reduce expression of TLR4 and enhance expression of TLR2, amongst the generated postbiotic mixtures. Regarding female sex hormones, it is well known that estrogens and progesterone are master regulators of the immune system and mucosal barrier in the female reproductive tract (Peters et al., 2022). These hormones may be similarly important in the gastrointestinal tract, with their reduction observed during menopause reducing barrier integrity and increasing microbial translocation in the gut as well. Accumulating evidence has linked TLR function to estrogen and estrogen receptor α (ERα) and β (ERβ), suggesting a possible contribution of different microbial sensing and TLR activation to sex bias in chronic inflammatory diseases, likely linked to gut MAMPs recognition (Maffei et al., 2022; Peters et al., 2022). Experimental evidence indicates that estradiol and progesterone maintain the gut barrier and protect from gut injury. Recent studies have reported that intestines from female rat are more resistant to shock-induced injury than from male rat, with the resilience being attributed to estradiol levels (Maffei et al., 2022). *In vitro* treatment with estradiol protects mucus-producing intestinal epithelial cells against oxidant injury (Homma et al., 2005), while colonic epithelial barrier function is protected through the estrogen receptor-β signaling (Langen et al., 2011; Diebel et al., 2015). Both estradiol and progesterone improve epithelial barrier function by upregulating tight junction proteins (Braniste et al., 2009). Estrogen mRNA has been found reduced in the colon of animal with colitis and patients with inflammatory bowel disease (Collins et al., 2017). Plasma progesterone levels have been inversely correlated with plasma lipopolysaccharide, a marker of reduced gut barrier function (Zhou et al., 2018). The results of this study suggest that VMK223 has the potential to reduce markers of systemic inflammation in healthy females over 40 years old. VMK223 is a mixture of *B. breve* derived MAMPs that act as TLR4 antagonist and TLR2 agonist *in vitro*, and the supplementation of VMK223 is followed by significant improvements in the level of specific sex hormones, the depletion of which during the perimenopause and menopause stage increases gut permeability and systemic inflammation. Taken together the results suggest that VMK223 may protect intestinal epithelial barrier function limiting gut permeability and preventing microbial translocation. When the MAMPs in VMK223 (CPS and EPS from *B. breve*) interact with TLR2 and TLR4 on the intestinal epithelium, they activate certain proteins and transcription factors that enhance the function of the intestinal epithelial barrier, reducing intestinal permeability. This may prevent microbial translocation and gut microbiome metabolites to trigger an immune response, based on the reduction of cortisol levels, preventing thus the accumulation of systemic inflammation that eventually can lead to chronic inflammatory states. The potential mechanism, however, has not been evaluated in this study, and future studies focusing on markers of intestinal epithelial barrier function are warranted. Additionally, given that perimenopause can last 7–15 years with a wide range of impact on immune and endocrine function, future studies should consider adopting a longitudinal approach to monitor changes throughout this period, ideally by recruiting a larger sample.

## 5 Conclusion

The prospect of restoring a normal microbiota is an appealing hypothesis to manage inflammation mediated conditions associated with a gut microbiota dysbiosis. Restoration of the composition of gut microbiota and its community structure conceivably leads to restoration of its function. Several ways for manipulating gut microbiota composition and function include the use of dietary interventions based on probiotics and prebiotics. However, due to environmental factors such as diet, lifestyle and drugs being major determinants of gut microbiota composition and activity, the effectiveness of such approaches at population level is reduced. Postbiotics on the other hand, offer a promising alternative by delivering the actual bioactives of the probiotic bacteria, without depending on the presence and metabolic activity of the beneficial bacteria in the GI tract. In the current study, VMK223, a probiotic cell wall derived postbiotic, showed to improve immune and endocrine markers of intestinal epithelial barrier function. Further investigation into the immunomodulatory potential of cell wall components and their specific interactions with innate immune cells and TLRs holds promise for developing advanced therapeutics and understanding the precise molecular mechanisms underlying these interactions will contribute to the rational design of targeted interventions for various health conditions.

## Data availability statement

The original contributions presented in the study are included in the article/Supplementary material, further inquiries can be directed to the corresponding author.

## Ethics statement

The studies involving humans were approved by University of Roehampton Research Ethics Committee (Ethics reference number: LSC 18/274). The studies were conducted in accordance with the local legislation and institutional requirements. The participants provided their written informed consent to participate in this study.

## Author contributions

D-EM: Conceptualization, Investigation, Writing – review & editing, Data curation. BB: Investigation, Writing – review & editing. PH: Writing – review & editing. GT: Writing – review & editing. JV: Writing – review & editing. AC: Conceptualization, Formal analysis, Investigation, Methodology, Writing – original draft, Writing – review & editing, Data curation.

## Footnotes

^1^ http://clinicaltrials.gov
