# Supplementary material for: Supplementation with postbiotic from Bifidobacterium Breve BB091109 improves inflammatory status and endocrine function in healthy females: a randomized, double-blind, placebo-controlled, parallel-groups study
Source: Front Microbiol. 2023 Nov 23;14:1273861. doi: 10.3389/fmicb.2023.1273861 (PMC10702524; doi:10.3389/fmicb.2023.1273861)
Supplement: Supplementary file 1 [file Table_1.docx]

**TABLE S1. Screening of compounds on Human TLR2 & TLR4 expressing cell lines. Optical density (OD, 655nm) of secreted alkaline phosphatase (SEAP) production following 24h incubation at 37^o^C in a CO_2_ incubator. PAM2 and LPS K12 were used as ligands for human TLR2 and hTLR4 (Mean ± SD).**

|  | **Treatment** | | |
| --- | --- | --- | --- |
|  | **Ligand**  (positive control) | **VMK223**  (agonistic interaction) | **Ligand & VMK223**  (antagonistic interaction) |
| hTLR2 | 3.461 ± 0.102 | 1.939 ± 0.067 | 3.339 ± 0.209 |
| hTLR4 | 2.158 ± 0.128 | 0.088 ± 0.010 | 0.385 ± 0.024 |

*Human embryonic kidney 293 (HEK293) cells that stably express a functional TLR2 or TLR4 gene and a secreted alkaline phosphatase (SEAP) reporter gene were obtained from InvivoGen (HEK blue-4 cells; UK) and used to study the potential of VMK223 to act as a microbial associated molecular pattern for the specific toll like receptors, following the manufacturer’s instructions. Briefly, 20 μl of VMK223 (10 times diluted) was used in the absence (agonistic interaction) or presence (antagonistic interaction) of appropriate ligand (PAM2 (100 ng/ml) for the 293-hTLR2 cell line; LPS K12 (100 ng/ml) for the 293-hTLR4 cell line) to stimulate the cell lines in a 200μl of final reaction volume. The activation of the artificial NF-κB-inducible promoter and the subsequent production of SEAP was measured by reading the optical density (OD) at 655 nm. Samples and controls were tested in duplicate.*
